# Incident gabapentin prescribing associated with opioid and benzodiazepine/Z-drug prescribing – a population-based longitudinal study in primary care

**DOI:** 10.3389/fphar.2025.1583415

**Published:** 2025-08-13

**Authors:** Kristjan Linnet, Bjarni Pall Linnet Runolfsson, Johann Agust Sigurdsson, Larus Steinthor Gudmundsson

**Affiliations:** ^1^ Development Centre for Primary Healthcare in Iceland, Primary Healthcare of the Capital Area, Reykjavik, Iceland; ^2^ Faculty of Pharmaceutical Sciences, School of Health Sciences, University of Iceland, Reykjavik, Iceland; ^3^ General Practice Research Unit, Department of Public Health and Nursing, Norwegian University of Science and Technology (NTNU), Trondheim, Norway

**Keywords:** gabapentin, opioids, benzodiazepine/Z-drugs, long-term co-prescribing, primary care, ICD-10 conditions

## Abstract

**Aim:**

To investigate the association between long-term prescribing of opioids, benzodiazepines and Z-drugs, and the incidence of gabapentin prescribing.

**Methods:**

From January 2009 to December 2012, 219,800 patients contacted primary healthcare centres in the Reykjavik metropolitan area. Of these, 94,840 patients aged 10–69 years, met the inclusion criteria. Data on relevant ICD-10 conditions related to multimorbidity were retrieved from a comprehensive medical records database for the primary healthcare centres in the area run by the Primary Healthcare of the Capital Area. Information on redeemed prescriptions for the relevant drugs was obtained from the Medicines Registry of the Directory of Health. The subjects were divided into four groups based on long-term use of opioids, benzodiazepines (BZDs) and Z-drugs (Defined Daily Doses, DDDs). The incidence rate ratio (IRR) of gabapentin was assessed using Cox regression. The effect of certain chronic conditions and long-term use of selective serotonin reuptake inhibitors (SSRIs) was also explored. Finally, the amount and duration of future gabapentin use were examined.

**Results:**

Long-term use of opioids, BZDs or Z-drugs was associated with an increased risk of future gabapentin use. Individuals with the highest use of opioids and benzodiazepines or Z-drugs, i.e., ≥30 DDDs BZDs or Z-drugs were most likely to initiate gabapentin therapy (IRR 7.18: 95% CI 6.50–7.93). These individuals also had the longest average duration of future gabapentin use.

**Conclusion:**

The rise in gabapentin prescriptions must be carefully monitored as prior history of opioids, benzodiazepines and Z-drugs may pose a risk of problematic gabapentin use.

## Introduction

Gabapentin was approved in the United States in 1993 by the Food and Drug Administration (FDA) for the treatment of epilepsy and later as an analgesic for post-herpetic neuralgia (Peckham et al., 2018). It was approved the same year in the United Kingdom (Rahman et al., 2021) and in 2006, the European Medicines Agency (EMA) granted a marketing authorisation in all member states of the European Union for gabapentin for epilepsy in patients with partial seizures and neuropathic pain (European Medicines Agency - Committee for Medicinal Products for Human Use, 2006). Since then, the prescribing of gabapentin has increased substantially, which may be partly related to an increase in off-label use (Montastruc et al., 2018; Torrance et al., 2020; Costales and Goodin, 2021).

The clinical activity of gabapentin is thought to be due to a selective inhibitory effect on voltage-gated calcium channels containing the α_2_δ-1 subunit, thus reducing depolarization-induced calcium influx and subsequently increasing GABA in the brain, in addition to reducing neurotransmitter release. However, it remains to be determined whether this mechanism of action can sufficiently account for the broad clinical spectrum of the gabapentinoids (Sills, 2006). Gabapentin has been used in a variety of off-label indications where the lack of medications for the treatment of various types of pain may have played a role in its increased prescribing. It is relatively well tolerated over a broad dosage range which might have contributed to the view that off-label prescribing could be considered more or less safe. Initially gabapentin was thought to have no abuse potential. Later on, concerns were expressed about possible misuse, particularly in patients with other substance use disorders (SUD), especially opioid use disorder. Co-prescribing gabapentin with opioids and benzodiazepines (BZD) and benzodiazepine-related drugs (Z-drugs) may increase mortality (Smith et al., 2016).

The question of misuse is still debated, and we do not have any research work on this issue in Iceland. The increased use of gabapentin cannot be explained by the registered indications only so that points to off-label use, and part of it could be related to abuse or misuse. Research data is lacking so we have to point to international studies that can be found in the literature (Bastiaens et al., 2016; Grosshans et al., 2013; Smith et al., 2016).

In a study on overdose deaths involving non-BZD hypnotic/sedatives in the United States (Tardelli et al., 2022) the authors state that gabapentin and Z-drugs were introduced as less dangerous alternatives to benzodiazepines and opioids, thus creating perceptions of increased safety without formidable data to back it up, leading to increased prescribing of these drugs.

A study on multimodal analgesic therapy with gabapentin in patients undergoing laparoscopic surgery found it to be associated with increased rates of respiratory depression during phase-I recovery OR = 1.47 (95% CI. 1.22–1.76) (Cavalcante et al., 2017). It seems that GABA-mimetic dependence is usually mixed with use of other substances while pure GABA-dependence seems to be absent (Bonnet and McAnally, 2022). A Canadian study found that co-prescribing of gabapentin with opioids was associated with a considerable increase in the risk of opioid-related death, particularly at higher doses (Gomes et al., 2017). An American study found that across all participant cohorts the use of gabapentin combined with opioids and benzodiazepines was associated with significantly higher odds of respiratory depression and substance-related overdose (Olopoenia et al., 2022).

**FIGURE 1:**
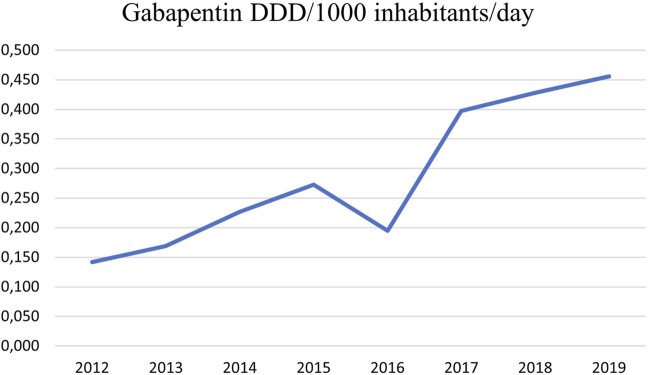
Prescriptions of gabapentin in primary care in the Reykjavik metropolitan area from 2012 to 2019 in DDDs/1,000 inhabitants/Day. It increased from 0.142 in 2012 to 0.456 in 2019. Information retrieved from the PHCCA medical records database.

The prescription of gabapentin has increased considerably in Iceland as in other European countries in recent years (Peckham et al., 2018; Rahman et al., 2021; Torrance et al., 2020). It increased from 0.142 DDDs/1,000 inhabitants/day in 2012 to 0.456 DDDs/1,000 inhabitants/day in 2019 (Figure 1). A longitudinal trend study in 65 countries and regions across the world showed that despite differences in healthcare system and culture a consistent increase in gabapentinoid consumption is observed worldwide (Chan et al., 2023). The aim of the study was to find the incidence of gabapentin prescribing in primary healthcare in Iceland and whether it was associated with long-term prescribing of benzodiazepines, Z-drugs and opioids, i.e., consistent use during at least three consecutive years. We looked at the distribution of several chronic diseases in the cohort using these drugs, and also long-term use of SSRIs to determine if these issues could possibly play a role as predictors of gabapentin use.

According to the Nordic Medico-Statistical Committee (NOMESCO) Icelanders are among the highest users of both SSRIs and opioids in Scandinavia. This has been pointed out by the Directorate of Health. It is not unlikely that off-label use is mostly to be found among younger patients and patients outside the primary healthcare, i.e., in psychiatry settings.

Pregabalin is registered in Iceland, but we chose to look at gabapentin in our study as it has been much longer on the market. A recently published Danish study reported that gabapentin and pregabalin were used equally (Pottegård et al., 2025). The populations in the Scandinavian countries are very similar and that applies both to Denmark and Iceland.

The use of gabapentin as pain medicine, has increased in Iceland as in a number of other countries (see Figure 1), but probably also in an attempt to reduce opioid prescribing (Tardelli et al., 2022). In our study it seems that gabapentin use is related to diseases where pain is a difficult health issue. It could be more deeply rooted, e.g., physical dependency and allostatic load which might play a role in this context. Allostatic load reflects the cumulative strain on the organism over time, while allostatic overload signifies a physiological state where adaptability is compromised, leading to a heightened risk of complex disease development.

## Materials and methods

### Design and setting

This retrospective study used data from a population of 219,800 persons who contacted healthcare centres within the Primary Healthcare of the Capital Area (PHCCA) as well as independent healthcare centres in the Reykjavik metropolitan area during a four-year period from 1 January 2009 to 31 December 2012. There are 15 healthcare centres within the PHCCA and four independent healthcare centres in the Reykjavik metropolitan area, totalling 19 healthcare centres. The primary healthcare centres are staffed by general practitioners, midwives, nurses and other personnel. The PHCCA runs a collective medical records database (called Saga-database) comprising medical records from all the primary healthcare centres in the metropolitan area. Information on the age and sex of all patients who contacted the healthcare centres during the aforementioned four-year period was retrieved from the PHCCA medical records database, as well as the diagnosis (International Statistical Classification of Diseases and Related Health Problems 10th Version; ICD-10) for chronic conditions as has been described in three previous papers (Linnet et al., 2016; Linnet et al., 2019; Linnet et al., 2022). The distribution of age and sex in the cohort is shown in Table 1, which presents the baseline characteristics of the participants. The data in the PHCCA medical records database are stored under a unique personal identifier (ID) allocated to every inhabitant.

**TABLE 1:** Baseline characteristics of the individuals in current study.

Characteristics	Group 1 No drug users^ǂ^	Group 2 Opioids & <30 DDD BZD/Z	Group 3 BZD/Z	Group 4 Opioids & ≥30 DDD BZD/Z
Number of persons	n = 82,251	n = 3,640	n = 6,354	n = 2,595
Men	41,564 (50.5%)	1,361 (40.0%)	2,197 (34.6%)	920 (35.5%)
Women	40,687 (49.5%)	2,045 (60.0%)	4,157 (65.4%)	1,675 (64.5%)
Mean age, years (±SD)	33.8 (15.8)	38.8 (15.3)	52.7 (11.6)	52.6 (11.8)
Fibromyalgia/Myalgia	18,915 (77.7%)	1,322 (5.5%)	2,907 (12.0%)	1,189 (4.8%)
Epilepsy	497 (70.4%)	18 (2.5%)	111 (15.8%)	79 (11.3%)
Mental disorders	17,671 (77.9%)	1,248 (5.5%)	2,789 (12.3%)	975 (4.3%)
RA	1,377 (70.7%)	121 (6.3%)	316 (16.3%)	131 (6.7%)
OA	2,302 (59.0%)	236 (6.2%)	955 (24.4%)	409 (10.4%)
Chronic back pain	16,249 (77.3%)	1,271 (6.0%)	2,432 (11.6%)	1,063 (5.1%)
Other conditions	34,930 (80.3%)	2,040 (4.7%)	4,635 (10.7%)	1,887 (4.3%)
SSRI use	2,091 (46.8%)	204 (4.5%)	1,408 (31.6%)	763 (17.1%)

^a^ Use neither opioids nor benzodiazepines/Z-drugs during the exposure window.

Abbreviations: RA, rheumatoid arthritis; OA, osteoarthritis; SSRI, selective serotonin reuptake inhibitors.

Other conditions, includes, asthma, bronchiectasis, tuberculosis, herpes zoster, human immunodeficiency virus, thyroid diseases, diabetes, metabolic diseases, hyperlipidaemia, cardiovascular disease, hypertension, chronic obstructive pulmonary disease, gastro-oesophageal reflux, psoriasis, ankylosing spondylitis, osteoporosis, other chronic musculoskeletal problems and renal disease.

### Exposure

The extracted data were linked through the patients’ IDs with data from the Icelandic Medicines Registry (IMR) on prescriptions for the drugs under investigation; gabapentin, opioids (codeine, tramadol and strong opioids), benzodiazepines and Z-drugs. All redeemed prescriptions for drugs with the Anatomical Therapeutic Chemical (ATC) codes N02A (Opioids), N02AA (Natural opium alkaloids), N02AA01 (Morphine), N02AB (Phenylpiperidine derivatives), N02AJ06 (Codeine and paracetamol), N02AX02 (Tramadol), N03AX12 (Gabapentin), N05BA (Anxiolyt. Benzodiazepine derivatives), N05CD (Hypnot. Sedat. Benzodiazepine derivatives), N05CF (Benzodiazepine related drugs), and N06AB (Selective serotonin reuptake inhibitors) were retrieved and collected in the study database. When all relevant data had been linked through the subjects’ IDs it was encrypted so the personal identity of the patients would not be revealed during the processing of the data set. This was done in accordance with the data protection standards required by law. The extracted data was kept at the Directory of Health without any accessibility for the researchers or any additional persons. The encrypted data set was handed over to the researchers for the analysis Exclusion criteria were based on the 219,800 individuals who visited all the primary healthcare centres of the capital area in Iceland from 2009 to 2018. Individuals younger than 10 years of age and older than 69 years of age were excluded. Very few in the study population under 10 years of age are prescribed gabapentin so it is reasonable to exclude them. Adding the participants 70 years of age and above could have an influence on the prevalence, but does not make any difference concerning the research question. Individuals with intermittent use of Z-drugs, benzodiazepines and opioids from January 2009 to December 2012 were also excluded from the data as they did not meet the criteria of long-term users. In this study the term intermittent user is applied to those who were prescribed drugs in the aforementioned drug classes occasionally during the four-year period for less than 3 consecutive years, whereas long-term users are those who used them consistently during at least three consecutive years. Individuals who died during the four-year interval between 2009 and 2012 were also excluded, along with cancer patients, leaving us with 94,840 participants. Persons who were prescribed Z-drugs, benzodiazepines or opioids were identified and were followed-up, beginning 3 years after the first prescription. We used the same definition of low, medium and high-dose users as in our previous paper (Linnet et al., 2019) where the upper limit for the low dose is three prescriptions per year according to clinical guidelines (4 weeks). We had information on all redeemed drugs but not on adherence. We assume that long-term patients who claimed their prescriptions on a regular basis took their drugs. We find it reasonable to expect that patients who redeem their prescriptions year after year through a long period most likely take their medications. Nevertheless, we do not know it for sure as we have not done any research work which can prove it.

### Covariates

In this paper the consecutive use of SSRIs during the four-year period 2009–2012 with a minimum of three-year continuous use is a relevant covariate. SSRIs are used as a proxy for mental disorders (F00-F99). Several chronic conditions pertaining to the definition of multimorbidity as reported in previous papers (Linnet et al., 2016; Linnet et al., 2019; Linnet et al., 2022) are also relevant. These conditions include F00-F99: mental disorders, G40: epilepsy, M05-M14: rheumatoid arthritis, M15-M19: osteoarthritis, M53-M54: chronic back pain, M79: fibromyalgia/myalgia. Additionally, 18 other chronic conditions were combined into one group of disease variables: other conditions (Supplementary Table S1), i.e. the remaining ICD-10 diagnoses.

The cohort was divided into four groups for the period 1 January 2009 to 31 December 2012 depending on the use of opioids, benzodiazepines and Z-drugs: Group 1 who neither used benzodiazepines, Z-drugs nor opioids during these years, Group 2 who continuously used opioids over a three-year period with low or minimal intermittent use of benzodiazepines and/or Z-drugs. Group 3 comprising individuals with a continuous three-year use of benzodiazepines and/or Z-drugs Group 4 including persons with a continuous three-year co-prescribing of opioids and benzodiazepines and/or Z-drugs during this period.

### Outcome

As mentioned earlier, information on redeemed prescriptions for the drugs under investigation were retrieved from the Icelandic Medicines Registry (IMR) and linked through the patients’ IDs to the ICD-10 diagnoses from the medical records. The primary outcomes of interest were the incident prescribing of gabapentin in patients who were regular users of opioids, benzodiazepines, and/or Z-drugs depending on different combination of the drugs and their dosages, comparing low and high dosages.

### Subject groups and statistical analysis

**FIGURE 2:**
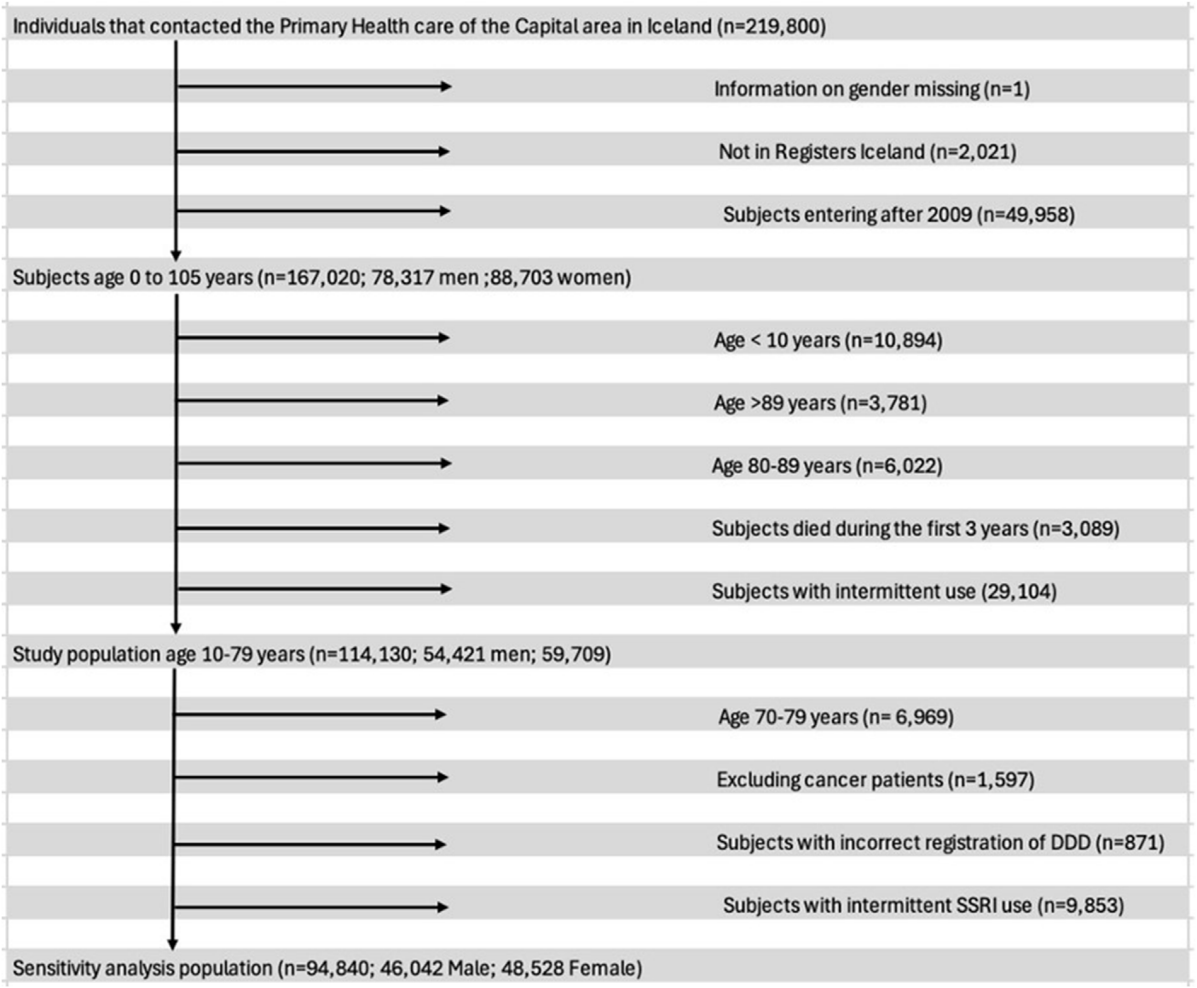
Flowchart of individuals included in the longitudinal cohort study in primary care.

The gabapentin use of primary care patients who were consistent users of opioids, benzodiazepines and/or Z-drugs during a three-year period from 1 January 2009 to 31 December 2012 was determined and compared with subjects who were not consistent users of these drugs. Participants in the study were between 10 and 69 years of age. Their age was recorded at entry. They were legal residents with domicile in Iceland, and thus health-insured. They attended primary healthcare centres in the Reykjavik metropolitan area during the three-year period from 2009 to 2012. A flowchart (Figure 2) shows the exclusions in our study. Among them were persons who sometimes used gabapentin with opioids, benzodiazepines and Z-drugs during the period 2009–2012 as well as persons with intermittent use of SSRIs but not within the definition of long-term use. After these exclusions we had a final cohort of 94,840 patients (10–69 years of age), 48% male and 52% female, who could be divided into four groups: (A) 82,251 persons who had neither been prescribed opioids, benzodiazepines nor Z-drugs for long-term use (B) 3,640 persons who had been prescribed opioids along with <30 DDDs of BZD/Z-drugs for long-term use and (C) 6,354 persons who had been prescribed both benzodiazepines and/or Z-drugs for long-term use and (D) 2,595 persons who had been prescribed opioids and ≥30 DDDs of benzodiazepines and Z-drugs per year for 3 consecutive years for long-term use.

Six different Cox regression models were conducted for analysis of the four different groups. For every model, adjustments were made for age and sex. Gabapentin incidence was determined as the frequency of gabapentin initiation after the long-term use of opioids, benzodiazepines or Z-drugs. The models were called; model 1a, model 1b and models 2–5. Model 1 was split into 1a and 1b. For 1a, adjustments were made for age, sex and fibromyalgia/myalgia, while epilepsy was added to model 1b. Mental disorders was added to model 2. In model 3, rheumatoid arthritis, osteoarthritis and chronic back pain were included. In model 4, seventeen other diagnoses of chronic conditions were combined as a single variable and added to that model. Finally, in model 5, long-term use of SSRIs was included.

Cox regression was used to determine the incidence rate ratio for the persons in the four groups with respect to future gabapentin use, while adjusting for age, sex and chronic diseases, along with long-term use of SSRIs. Group 1, the non-drug users were used as reference in every model. The 95% confidence intervals were used to evaluate statistical significance. Statistical analyses were conducted using R-studio software, version 2021.09.0 + 351. Excel version 16.71 was also used for making tables and figures for the analyses.

## Results

As previously noted, the individuals were divided into four groups with respect to their prior use of opioids, benzodiazepines and Z-drugs. This was done to determine if long-term use of these drugs would predict future gabapentin use. The inclusion of certain medical diagnoses was also of interest in predicting future gabapentin use. A total of 82,251 persons did not meet the criteria for long-term use of the drugs and were placed in Group 1, defined as non-drug users. In total 3,640 persons were long-term users of opioids (with codeine being most frequently used) and <30 DDDs of benzodiazepines or Z-drugs and were put in Group 2. In Group 3, there were 6,354 persons with a long-term use of BZD/Z-drugs, and in Group 4 there were 2,595 long-term users of opioids and ≥30 DDDs BZD/Z-drugs. The characteristics of the four groups of long-term users of Z-drugs, benzodiazepines or opioids are shown in Table 1.

Following exclusion of participants, the final number of subjects was 94,840 as seen in the flowchart in Figure 2 demonstrating the criteria used to exclude subjects from the analysis. Of those, 48,528 (52%) were women and the mean age was 33.8 years. The mean follow-up time was 862.5 days.

The simultaneous use of both opioids and benzodiazepines/Z-drugs (Group 4) proved to be the strongest predictor of future gabapentin use, with an Incidence rate ratio (IRR) of 7.18 (95% CI: 6.50–7.93) in models 1a (Fibromyalgia/Myalgia) and 1b (Epilepsy). Although the IRR decreased to 6.47 [95% CI: 5.84–7.17] in model 2 (Mental Disorders), it remained a major predictor of future use. Group 3, benzodiazepine or Z-drug users had the second highest IRR in models 1a, 1b and 2. The IRRs was almost the same for 1a and 1b, 5.20 (95% CI: 4.81–5.61) and 5.21 (95% CI: 4.82–5.63) respectively, while it lowered to 4.47 (95% CI: 4.10–4.87) for model 2. Group 2 (Opioids & <30 DDD BZD/Z-drugs) had the lowest IRR in models 1a, 1b and 2, although this group had over a threefold increase in gabapentin incidence compared to Group 1 (no drug use) in the models.

In models 1a and 1b, the incidence rate ratio was significantly higher in Group 4 (opioids & ≥30 DDD BZD/Z-drugs) for each of the three models (Table 2). The findings were statistically significant for fibromyalgia/myalgia and mental disorders. Epilepsy was the only covariate that did not have a statistically significant effect on the incidence of gabapentin prescribing with an IRR of 0.85 (95%CI: 0.64–1.13). In those models, adjustments were made for age and sex as in the baseline model where women had a higher incidence rate ratio than men, IRR 1.64 (95%CI: 1.52–1.76). The models show that long-term use of opioids, benzodiazepines and Z-drugs increase the risk of incident gabapentin use for every group together with fibromyalgia/myalgia and mental disorders. The incidence rate ratio for epilepsy shows that this disease does not increase the risk, with an IRR for Model 1b: 0.85 (95%CI: 0.64–1.13) and an IRR for Model 2: 0.82 (95%CI: 0.62–1.09).

**TABLE 2:** Incident gabapentin prescribing in relation to prior opioid and benzodiazepine/Z-drug use and co-morbidity.

Model 1a Fibromyalgia – Myalgia					Model 1b Epilepsy		Model 2 Mental disorders		Model 3 RA, OA & chronic back pain		Model 4 Other diseases		Model 5 SSRI use	
Groups	n = 94,840	Gabapentin incidence	IRR	95% CI	IRR	95% CI	IRR	95% CI	IRR	95% CI	IRR	95% CI	IRR	95% CI
Group 1 No drug users	82,251	1,987	1.0	REF	1.0	REF	1.0	REF	1.0	REF	1.0	REF	1.0	REF
Group 2 Opioids & < 30 DDD BZD/Z	3,640	305	3.26	2.88–3.69	3.25	2.87–3.69	3.19	2.82–3.62	3.00	2.65–3.40	2.94	2.60–3.34	2.92	2.57–3.31
Group 3 BZD/Z	6,354	1,065	5.20	4.81–5.61	5.21	4.82–5.63	4.47	4.10–4.87	4.12	3.78–4.50	4.08	3.74–4.45	3.86	3.53–4.22
Group 4 Opioids & ≥30 DDD BZD/Z	2,595	526	7.18	6.50–7.93	7.18	6.50–7.93	6.47	5.84–7.17	5.84	5.26–6.48	5.73	5.16–6.37	5.58	5.02–6.20
Fibromyalgia/myalgia	24,333	2,731	1.90	1.77–2.02	1.89	1.76–2.02	1.77	1.65–1.90	1.56	1.49–1.71	1.54	1.43–1.65	1.53	1.42–1.64
Epilepsy	705	66			0.85	0.64–1.13	0.82	0.62–1.09	0.81	0.62–1.08	0.81	0.61–1.07	0.78	0.57–1.03
Mental disorders	22,683	2,788							1.30	1.20–1.40	1.26	1.17–1.37	1.22	1.13–1.33
RA	1,945	333							1.23	1.08–1.41	1.21	1.06–1.38	1.21	1.06–1.39
OA	3,902	815							1.47	1.34–1.61	1.43	1.30–1.57	1.42	1.30–1.56
Chronic back pain	21,015	2,297							1.34	1.25–1.43	1.30	1.21–1.39	1.30	1.21–1.39
Other conditions	43,492	3,674									1.21	1.11–1.32	1.21	1.11–1.32
SSRI use	4,466	873											1.30	1.19–1.43

The incidence rate ratio (IRR) is calculated with the no drug group as a reference group. No drug means neither on opioids nor BZD/Z during the exposure window. The mean follow-up time was 1,724 days, SD was 779 days and the median follow-up time was 1,787 days (range, 1–1,826 days). Model 1a: Adjusted for age, sex and subjects with fibromyalgia/myalgia adjusted for opioids and BZD/Z, with exclusion of cancer patients (n = 94,840). The incidence of gabapentin initiation was 4,959 individuals during follow-up. Model 1. b: The same adjustments as for model 1a with the addition of epilepsy patients. Model 2: The same adjustments as for the previous models, adding subjects with mental disorders. Model 3: Similarly adjusted for age and sex, and also subjects with rheumatoid arthritis (RA), osteoarthritis (OA) and chronic back pain, adjusted as previously for opioids and BZD/Z-drugs, with exclusion of cancer patients (n = 94,840). Gabapentin incidence was 4,959 persons during follow-up. Model 4: The same adjustments as for model 3 with the addition of subjects suffering from 18 other diseases. Model 5: The same adjustments as for previous models, adding subjects with long-term SSRI use.

In Group 4, opioids with ≥30 DDDs BZD/Z-drugs, the IRRs were again highest for models 3 (RA, OA and chronic back pain), model 4 (Other) and model 5 (long term SSRI use). The IRR for group 4 in model 3 with the additions of the covariates, rheumatoid arthritis, osteoarthritis and chronic back pain was 5.84 (95%CI: 5.26–6.48), a strong indicator for future gabapentin use. Therefore, the combined use of opioids and ≤30 DDDs of benzodiazepines or Z-drugs is still the strongest predictor of future gabapentin use. The IRRs in those models decreased with the addition of other diagnoses, to 5.73 (95%CI: 5.16–6.37) and long-term SSRI use but were still over 5.50 (95%CI: 5.02–6.20). For Group 3 (benzodiazepine or Z-drug users) the IRR was 4.12 (95%CI: 3.78–4.50) in model 3 (with RA, OA and chronic back pain) and decreased to 4.08 (95%CI: 3.74–4.45) in model 4 (with 17 other diagnoses) and to 3.86 (95%CI: 3.53–4.22) for model 5 (with the addition of long-term SSRI). Group 2 (opioids & <30 DDD of benzodiazepines or Z-drugs) had the lowest IRRs ranging from 3.00 (95%CI: 2.65–3.40) to 2.92 (95%CI: 2.57–3.31) in models 3–5. The data suggests that epilepsy does not influence future gabapentin use, while the other conditions have a significant impact with osteoarthritis being the strongest predictor with an IRR of 1.47 (95%CI: 1.34–1.61) in model 3 (Table 2). In Table 3 we report the incident gabapentin prescribing in relation to low and high doses of opioids and Benzodiazepine/Z-drugs.

**TABLE 3:** Incident gabapentin prescribing in relation to the amount of opioids and benzodiazepine/Z-drugs prescribed during the exposure window.

Model 1a Fibromyalgia - myalgia					Model 1b Epilepsy		Model 2 Mental disorders		Model 3 RA, OA & chronic back pain		Model 4 Other diseases		Model 5 SSRI use	
Groups	n = 94,840	Gabapentin incidence	IRR	95% CI	IRR	95% CI	IRR	95% CI	IRR	95% CI	IRR	95% CI	IRR	95% CI
Low dose														
Group 1 No drug users	82,251	1,987	1.0	REF	1.0	REF	1.0	REF	1.0	REF	1.0	REF	1.0	REF
Group 2 Opioids ≤300 & BZD/Z < 30 DDDs	1,748	169	3.32	1.38–7.90	3.32	2.38–7.99	3.07	1.27–7.40	2.87	1.19–6.90	2.96	1.23–7.13	3.02	1.25–7.27
Group 3 BZD/Z ≤ 1,000	4,065	585	3.19	2.76–3.69	3.19	2.76–3.69	2.49	2.14–2.89	2.20	1.88–2.56	2.15	1.84–2.51	1.95	1.67–2.28
Group 4 Opioids ≤300 & BZD/Z ≤ 1,000 DDDs	1,987	437	2.17	1.54–4.80	2.71	1.53–4.80	2.57	1.46–4.55	2.46	1.40–4.36	2.43	1.38–4.30	2.45	1.39–4.34
High dose														
Group 2 Opioids >300 & BZD/Z ≥ 30 DDDs	1,622	136	5.01	4.42–5.67	5.01	4.43–5.68	4.03	3.54–4.59	3.37	2.95–3.85	3.30	2.89–3.77	3.04	2.65–3.48
Group 3 BZD/Z > 1,000	2,289	480	2.94	1.98–4.37	2.94	1.98–4.37	2.24	1.50.3.33	1.96	1.31–2.92	1.91	1.28–2.85	1.63	1.09–2.43
Group 4 Opioids >300 & BZD/Z > 1,000 DDDs	606	89	7.41	6.32–8.67	7.42	6.34–8.70	5.99	5.10–7.04	4.83	4.08–5.71	4.80	4.05–5.66	4.43	3.75–5.25
Fibromyalgia/Myalgia	24,333	2,731	2.21	2.04–2.39	2.21	2.04–2.39	2.01	1.85–2.18	1.77	1.03–1.92	1.66	1.53–1.81	1.65	1.52–1.80
Epilepsy	705	66			0.94	0.69–1.29	0.88	0.64–1.20	0.87	0.64–1.19	0.86	0.63–1.18	0.83	0.61–1.14
Mental disorders	22,683	2,788					1.65	1.51–1.80	1.55	1.42–1.69	1.49	1.37–1.63	1.41	1.29–1.54
RA	1,945	333							1.09	0.92–1.29	1.05	0.89–1.25	1.08	0.91–1.28
OA	3,902	815							1.65	1.47–1.84	1.57	1.40–1.76	1.55	1.38–1.74
Chronic back pain	21,015	2,297							1.50	1.38–1.62	1.42	1.31–1.54	1.42	1.30–1.54
Other	43,492	3,674									1.37	1.25–1.51	1.37	1.25–1.51
SSRI use	4,466	873											1.56	1.39–1.74

The incidence rate ratio (IRR) is calculated with the no drug group as a reference group. No drug means neither on opioids nor BZD/Z-drugs during the exposure window. The mean follow-up time was 1,724 days, SD was 779 days, and the median follow-up time was 1,787 days (range, 1–1,826 days). Model 1a: Adjusted for age, sex and subjects with fibromyalgia/myalgia, adjusted for opioids and BZD/Z-drugs, with an exclusion of cancer patients (n = 94,840), and two subgroups for groups 2–4 based on low or high defined daily doses (DDDs). Gabapentin incidence was 4,959 during follow-up. Model 1. b: The same adjustments as for model 1a with the addition of epilepsy patients. Model 2: The same adjustments as for previous models, adding subjects with mental disorders. Model 3: The same adjustments as for previous models with the addition of subjects with rheumatoid arthritis (RA), osteoarthritis (OA) and chronic back pain. Model 4: The same adjustments as for models 1-3 with the addition of subjects suffering from 18 other diseases. Model 5: The same adjustments as for previous models, adding subjects with a long-term SSRI use.

Other conditions, includes, asthma, bronchiectasis, tuberculosis, herpes zoster, human immunodeficiency virus, thyroid diseases, diabetes, metabolic diseases, hyperlipidaemia, cardiovascular disease, hypertension, chronic obstructive pulmonary disease, gastro-oesophageal reflux, psoriasis, ankylosing spondylitis, osteoporosis, other chronic musculoskeletal problems and renal disease.

## Discussion

### Principal findings

Our findings suggest that the greatest likelihood of incident gabapentin use is in individuals who simultaneously use opioids and ≥30 DDDs of benzodiazepines or Z-drugs (Group 4). Those with long-term use of benzodiazepines or Z-drugs without opioids (Group 3) were also three to five times more likely to have incident gabapentin use, while opioids and <30 DDDs of benzodiazepines or Z-drugs (Group 2) had the lowest incidence out of the three groups compared to non-drug users.

Of the chronic conditions, fibromyalgia/myalgia, mental disorders and osteoarthritis were the strongest predictors of incident gabapentin use. In the latter part of the analysis, the same diagnoses, with the inclusion of long-term SSRI use, were the strongest predictors of future gabapentin use.

A United Kingdom study reported a three-fold higher incidence of gabapentin prescribing in patients with osteoarthritis than in matched controls, and upon being prescribed a gabapentinoid, osteoarthritis patients had a 30% increased risk of subsequent gabapentinoid-opioid co-prescribing events (Dahai et al., 2021). In our study osteoarthritis along with fibromyalgia/myalgia and mental disorders had the largest impact of the chronic conditions on future gabapentin use.

A Scottish study reported a fourfold increase in gabapentin prescriptions from 2006-2016, and that co-prescribing was common, with almost 60% of those receiving gabapentinoids also being prescribed an opioid, a benzodiazepine or both in 2016 (Torrance et al., 2020).

An increase has also been reported in recent years in co-prescriptions of gabapentin and Z-drugs as they have been considered a safer option than benzodiazepines and opioids (Tardelli et al., 2022). Finally, the findings show that women were at a 64% greater risk of incident gabapentin use than men. From 2016 to 2019, incident gabapentin prescriptions increased steadily in the United Kingdom, where the drug was most commonly prescribed with opioids, antidepressants, benzodiazepine and Z-drugs (Ashworth et al., 2023).

Group 2 comprised subjects who had a history of long-term opioid use together with low or minimal intermittent use of benzodiazepines and/or Z-drugs. The results show that the long-term users in Group 2 had an increased risk of incident gabapentin use.

When adding the diagnoses of fibromyalgia, epilepsy and mental disorders, the IRR decreased but was still threefold greater than for non-users of the aforementioned drugs. This finding remained stable when adding osteoarthritis, rheumatoid arthritis, chronic back pain and “other diseases” (Supplementary Table S1) and long-term SSRI use to the model, yielding an IRR for gabapentin use of 2.92 (95% CI: 2.57–3.31). Gabapentin is often prescribed as an off-label drug in the treatment of benzodiazepine withdrawal (Leung et al., 2022). This remains a concern as concomitant use of benzodiazepines and gabapentin increases the likelihood of misuse (Olopoenia et al., 2022). This also applies to concurrent use of opioids and gabapentin, as patients with opioid dependence syndrome tend to misuse gabapentin (Grosshans et al., 2013). It is therefore important to review prior use of opioids, benzodiazepines and Z-drugs before prescribing gabapentin to individuals prescribed these drugs as they are vulnerable to concomitant use and more likely to engage in problematic use as described in other studies (Hahn et al., 2022).

When adding the other variables, the risk decreased to fivefold in models 1a (fibromyalgia), model 1b (epilepsy) and model 2 (mental disorders). The risk decreased to fourfold for model 3 (osteoarthritis, rheumatoid arthritis, chronic back pain) and model 4 (“other diseases”) and just under fourfold in model 5 (with the addition of long-term SSRI use). Long-term users in Group 3 were therefore at an increased risk of incident gabapentin prescription, independent of chronic conditions or long-term use of SSRI. This places these individuals at an increased risk of abusing gabapentin. Previous studies suggest that combining gabapentin with benzodiazepines or Z-drugs is common and can have fatal consequences (Cho et al., 2020; Kaufmann et al., 2017). When subjects with a history of benzodiazepine use begin taking gabapentin, they do not necessarily stop using benzodiazepines (Bramness et al., 2010). Thus, initiation of gabapentin therapy in this group needs to be monitored carefully.

The subjects in group 4 (opioids & ≥30 DDDs of benzodiazepines or Z-drugs) had more than a sevenfold increase in risk of future gabapentin use. As for the other groups, the IRR decreased slightly when adding the other variables to the model. The risk remained more than fivefold for subjects in that group. Individuals in Group 4 are therefore more prone to incident gabapentin use than the subjects in the other groups. This might serve as a predictor of misuse and addiction as concomitant use of opioids and benzodiazepines or Z-drugs with gabapentin is often used to reach a euphoric state (Iacobucci, 2017). Recent research has also addressed the increase in mortality rate among individuals that simultaneously abuse opioids and benzodiazepines or Z-drugs with gabapentin (Bonnet and Scherbaum, 2017). The co-prescription of opioids and gabapentin increases the likelihood of opioid overdose when compared to individuals who only received opioid therapy (Gomes et al., 2017). A newly published study found that prescription of gabapentinoids and Z-drugs is common among opioid-agonist treatment patients and that co-prescription is associated with increased risk of drug-related deaths (Glancy et al., 2024).

### Low and high doses

We investigated whether the association between prescribing gabapentin on one side and opioids and benzo/Z-drugs on the other side was influenced by the dosages of these drugs, and whether there was an association between their dosages and the length of gabapentin use. The findings regarding long-term use of low doses of opioids (≤300 DDDs & >30 DDDs of BZD/Z) in Group 2 show over a threefold increase in the likelihood of incident gabapentin use. Subjects with high DDDs (>300 & ≥30 DDDs of BZD/Z) were found to have increased risk compared to the low-dose group, although this difference cannot be confirmed as significant because the intervals for the two subgroups overlap. In Group 3, subjects in the low-dose group (≤1,000 DDDs of BZD/Z) were at higher risk of incident gabapentin prescription than those who had used high doses of benzodiazepines or Z-drugs. The individuals in the low-dose group had a threefold increase in risk of future gabapentin use that decreased to approximately twofold in the final model. In Group 4, the low-dose subgroup (≤300 and BZD/Z ≤1,000) had greater than a twofold increase in risk in all models, while subjects in the higher-dose group (>300 DDDs of opioids and >1,000 of BZD/Z) had between a four-to sevenfold increase in risk. This makes Group 4 with high DDDs the group most prone to incident gabapentin use. Earlier studies have shown that higher doses of benzodiazepines or gabapentin along with opioid use increase the risk of opioid-related death (Chen et al., 2022). These findings highlight the importance of avoiding concomitant opioid and gabapentin or benzodiazepine use. We do not report more of these results here but point to Table 3.

When adding 18 chronic conditions to the models, the IRR of gabapentin prescription for the groups decreased slightly compared to the baseline model. Thus, prior use of the drugs was still the strongest predictor, even after the addition of the chronic conditions. Long-term use of SSRI drugs was also a strong predictor of initiation of gabapentin therapy, which became stronger in the dose-dependent models. In the last model, the subjects with a history of long-term use of SSRI drugs showed >50% increase in future gabapentin use which is consistent with the results of previous studies showing that gabapentin is often prescribed as an off-label drug for individuals suffering from specific mental health disorders (Dooley et al., 2002; Bastiaens et al., 2016). This is also the case for model 2 that includes a diagnosis of mental disorder within the low and high subgroups. Subjects with mental disorders were found to have a 65% increase in future gabapentin use. This increases the likelihood of gabapentin exposure to the patient population (Bastiaens et al., 2016). The diagnoses of mental disorders do not provide sufficient evidence regarding specific mental health disorders, such as alcohol withdrawal syndrome or bipolar disorder, for which individuals are frequently prescribed gabapentin. Thus, the mental disorders are a strong predictor of incident gabapentin use, but missing information about specific diagnoses precludes a meaningful interpretation of this finding. Other diagnoses also give a strong prediction of future gabapentin use with fibromyalgia and osteoarthritis being the strongest predictors. Gabapentin is prescribed for neuropathic pain, but results for fibromyalgia are limited (Giorgi et al., 2024). In the United Kingdom, gabapentin prescribing in patients with osteoarthritis has increased markedly while evidence for a benefit of the treatment is lacking (Appleyard et al., 2019). The results of this study are therefore unexpected when it comes to incident gabapentin use among individuals with osteoarthritis. Subjects with chronic back pain have a 30%–50% increase in incident gabapentin prescription, while subjects suffering from 18 other chronic conditions, such as asthma, tuberculosis, herpes zoster, human immunodeficiency virus infection, thyroid diseases and diabetes have 20%–37% increase in incident gabapentin use. It was surprising to see that epilepsy, a disease for which gabapentin was originally approved as a treatment option in the 1990s, was not associated with increased risk for future gabapentin use. On the other hand, patients with rheumatoid arthritis had a 20% increase in the risk of future gabapentin use.

### Strengths and limitations

This is a comprehensive study covering approximately two-thirds of the total population where all contacts with all the primary healthcare centres in the area are registered in the same medical records database. The distribution according to age and sex is the same as for the population at large making the cohort quite representative. By linking the IDs to the data on redeemed prescriptions in the Icelandic Medicines Registry during the study period we were able to cover all prescriptions for the relevant drugs issued by the GPs and claimed by patients in the Reykjavik metropolitan area. The length of the period of long-term prescribing, i.e., consecutive use of the relevant drugs for a minimum of 3 years can be seen as a strength.

As to be expected the study has some limitations, including the retrospective design. Since we did not find any population-based studies on association between current diagnoses, use of hypnotics, sedatives and opioids in relation to incident gabapentin use, we presented the analyses in a stepwise manner, without dropping any variables. This will help comparison of variables and their effects in studies on incident gabapentin use from other cohorts. As mentioned in the introduction chapter there are several reasons for the prescribing of gabapentin in primary care. This study material did not allow further analysis of the specific reasons for its prescriptions. Furthermore, information about specific diagnoses within the mental disorder category was lacking as the study database only included the merged ICD-10 category F00-F99. As gabapentin is frequently prescribed as an off-label drug for individuals suffering from alcohol withdrawal syndrome, anxiety disorders and bipolar disorder, it warrants a further study to explore the effect of these conditions on gabapentin use.

## Conclusion

Long-term use of opioids, benzodiazepines and Z-drugs increases the likelihood of initiation of gabapentin prescribing which might become problematic. The Incidence Rate Ratios revealed in this study can be useful for clinicians who should assess the risk-benefit balance of this drug combination before they decide to prescribe gabapentin for patients using these drugs.

## Data Availability

The data is retrieved from a medical records database in the Primary Healthcare of the Capital Area, and from the Icelandic Medicines Registry of the Directorate of Health, and is not publicly available. The encrypted data is kept at the Directorate of Health and can be made available on a reasonable request if permitted by the above-mentioned health authorities. Requests to access the databases should be directed to www.landlaeknir.is and visindanefnd@heilsugaeslan.is.

## References

[B1] Appleyard T. Ashworth J. Bedson J. Yu D. Peat G. (2019). Trends in gabapentinoid prescribing in patients with osteoarthritis: a United Kingdom national cohort study in primary care. Osteoarthr. Cartil. 27 (10), 1437–1444. 10.1016/j.joca.2019.06.008 31276819

[B2] Ashworth J. Bajpai R. Muller S. Bailey H. T. Harrison S. A. Whittle R. (2023). Trends in gabapentinoid prescribing in UK primary care using the clinical practice research datalink: an observational study. Lancet Reg. Health Eur. 27, 100579. 10.1016/j.lanepe.2022.100579 37069852 PMC10105252

[B3] Bastiaens L. Galus J. Mazur C. (2016). Abuse of gabapentin is associated with opioid addiction. Psychiatr. Q. 87 (4), 763–767. 10.1007/s11126-016-9421-7 26887855

[B4] Bonnet U. McAnally H. B. (2022). How prevalent and severe is addiction on GABAmimetic drugs in an elderly German general hospital population? Focus on gabapentinoids, benzodiazepines, and z-hypnotic drugs. Hum. Psychopharmacol. Clin. Exp. 37 (3), e2822. 10.1002/hup.2822 34687489

[B5] Bonnet U. Scherbaum N. (2017). How addictive are gabapentin and pregabalin: a systematic review. Eur. Neuropsychopharmacol. 27 (12), 1185–1215. 10.1016/j.euroneuro.2017.08.430 28988943

[B6] Bramness J. G. Sandvik P. Engeland A. Skurtveit S. (2010). Does pregabalin (Lyrica®) help patients reduce their use of benzodiazepines? A comparison with gabapentin using the Norwegian prescription database. Basic Clin. Pharmacol. Toxicol. 107 (5), 883–886. 10.1111/j.1742-7843.2010.00590.x 22545971

[B7] Cavalcante A. N. Sprung J. Schroeder D. R. Weingarten T. N. (2017). Multimodal analgesic therapy with gabapentin and its association with postoperative respiratory depression. Anesth. Analg. 125 (1), 141–146. 10.1213/ANE.0000000000001719 27984223

[B8] Chan A. Y. L. Yuen A. S. C. Tsai D. H. T. Lau W. C. Y. Jani Y. H. Hsia Y. (2023). Gabapentinoid consumption in 65 countries and regions from 2008 to 2018: a longitudinal trend study. Nat. Commun. 14, 5005. 10.1038/s41467-023-40637-8 37591833 PMC10435503

[B9] Chen T. C. Knaggs R. D. Chen L. C. (2022). Association between opioid-related deaths and persistent opioid prescribing in primary care in England: a nested case-control study. Br. J. Clin. Pharmacol. 88 (2), 798–809. 10.1111/bcp.15028 34371521

[B10] Cho J. Spence M. M. Niu F. Hui R. L. Gray P. Steinberg S. (2020). Risk of overdose with exposure to prescription opioids, benzodiazepines, and non-benzodiazepine sedative-hypnotics in adults: a retrospective cohort study. J. Gen. Intern Med. 35 (3), 696–703. 10.1007/s11606-019-05545-y 31919729 PMC7080944

[B11] Costales B. Goodin A. J. (2021). Outpatient off-label gabapentin use for psychiatric indications among US adults, 2011-2016. Psych. Serv. 72, 1246–1253. 10.1176/appi.ps.202000338 34015964

[B12] Dahai Y. Appleyard T. Cottrell P. G. (2021). Co-prescription of gabapentinoids and opioids among adults with and without osteoarthritis in the United Kingdom between 1995 and 2017. Rheumatology 60 (4), 1942–19517. 10.1093/rheumatology/keaa586 33159800

[B13] Dooley D. J. Donovan C. M. Meder W. P. Whetzel S. Z. (2002). Preferential action of gabapentin and pregabalin at P/Q-type voltage-sensitive calcium channels: inhibition of K+-evoked [3H]-norepinephrine release from rat neocortical slices. Synapse 45 (3), 171–190. 10.1002/syn.10094 12112396

[B14] European Medicines Agency (EMA) - Committee for Medicinal Products for Human Use (CHMP) (2006). Ref. Opin. Purs. article 30 Counc. Dir. 2001/83/EC Neurontin Assoc. names. 4–8-2006. Available online at: https://www.ema.euroa.eu/en/medicines/human/referrals/neurontin

[B15] Giorgi V. Sarzi-Puttini P. Pellegrino G. Sirotti S. Atzeni F. Alciati A. (2024). Pharmacological treatment of fibromyalgia syndrome: a practice-based review. Curr. Pain Headache Rep. 28, 1349–1363. 10.1007/s11916-024-01277-9 39042299 PMC11666752

[B16] Glancy M. Palmateer N. Yeung A. Hickman M. Macleod J. Bishop J. (2024). Risk of drug-related death associated with co-prescribing of gabapentinoids and Z-drugs among people receiving opioid-agonist treatment: a national retrospective cohort study. Psychiat Res. 339, 116028. 10.1016/j.psychres.2024.116028 38917674

[B17] Gomes T. Juurlink D. N. Antoniou T. Mamdani M. M. Paterson J. M. van den Brink W. (2017). Gabapentin, opioids, and the risk of opioid-related death: a population-based nested case-control study. PLOS Med. 14 (10), e1002396. 10.1371/journal.pmed.1002396 28972983 PMC5626029

[B18] Grosshans M. Lemenager T. Vollmert C. Kaemmerer N. Schreiner R. Mutschler J. (2013). Pregabalin abuse among opiate addicted patients. Eur. J. Clin. Pharmacol. 69 (12), 2021–2025. 10.1007/s00228-013-1578-5 23989299

[B19] Hahn J. Jo Y. Yoo S. H. Shin J. Yu Y. M. Ah Y.-M. (2022). Risk of major adverse events associated with gabapentinoid and opioid combination therapy: a systematic review and meta-analysis. Front. Pharmacol. 13, 1009950. 10.3389/fphar.2022.1009950 36304170 PMC9593000

[B20] Iacobucci G. (2017). UK government to reclassify pregabalin and gabapentin after rise in deaths. BMJ 358, j4441. 10.1136/bmj.j4441 28947423

[B21] Kaufmann C. N. Spira A. P. Alexander G. C. Rutkow L. Mojtabai R. (2017). Emergency department visits involving benzodiazepines and non-benzodiazepine receptor agonists. Am. J. Emerg. Med. 35 (10), 1414–1419. 10.1016/j.ajem.2017.04.023 28476551 PMC5623103

[B22] Leung E. Ngo D. H. Espinoza J. A. Jr Beal L. L. Chang C. Baris D. A. (2022). A retrospective study of the adjunctive use of gabapentin with benzodiazepines for the treatment of benzodiazepine withdrawal. J. Psychiatr. Pract. 28 (4), 310–318. 10.1097/PRA.0000000000000639 35797687

[B23] Linnet K. Gudmundsson L. S. Birgisdottir F. G. Sigurdsson E. L. Johannsson T. M. O. Sigurdsson J. A. (2016). Multimorbidity and use of hypnotic and anxiolytic drugs: cross-sectional and follow-up study in primary healthcare in Iceland. BMC Fam. Pract. 17, 69. 10.1186/s12875-016-0469-0 27267943 PMC4896036

[B24] Linnet K. Sigurdsson J. A. Tomasdottir M. O. Sigurdsson E. L. Gudmundsson L. S. (2019). Association between prescription of hypnotics/anxiolytics and mortality in multimorbid and non-multimorbid patients: a longitudinal cohort study in primary care. BMJ Open 9, e033545. 10.1136/bmjopen-2019-033545 PMC6924757 31811011

[B25] Linnet K. Thorsteinsdottir H. S. Sigurdsson J. A. Sigurdsson E. L. Gudmundsson L. S. (2022). Co-prescribing of opioids and benzodiazepines/Z-drugs associated with all-cause mortality – a population-based longitudinal study in primary care with weak opioids most commonly prescribed. Front. Pharmacol. 13, 932380. 10.3389/fphar.2022.932380 36147347 PMC9485885

[B26] Montastruc F. Loo S. Y. Renoux C. (2018). Trends in first gabapentin and pregabalin prescriptions in primary care in the United Kingdom 1993-2017. JAMA 320 (20), 2149–2151. 10.1001/jama.2018.12358 30480717 PMC6583557

[B27] Olopoenia A. Camelo-Castillo W. Qata D. M. Adekoya A. Palumba F. Sera L. (2022). Patterns of opioid and benzodiazepine use with gabapentin among disabled Medicare beneficiaries – a retrospective cohort study. Drug Alcohol Depend. 230, 1091800. 10.1016/j.drugalcdep.2021.109180 34847506

[B28] Peckham A. M. Evoy K. E. Ochs L. Covvey J. R. (2018). Gabapentin for off-label use: evidence-Based or cause for concern? Subst. Abuse Res. Treatm 12, 1178221818801311–1178221818801318. 10.1177/1178221818801311 PMC6153543 30262984

[B29] Pottegård A. Rasmussen L. Olesen M. Sørensen A. M. S. Ennis Z. N. Kane J. (2025). Trends in gabapentinoid prescribing: a nationwide Danish drug utilization study. Brit J. Clin. Pharmacol. 10.1002/bcp.70060 PMC12381606 40205787

[B30] Rahman A. Kane J. Montastruc F. Renoux C. (2021). Trends in new prescription of gabapentinoids and of coprescription with opioids in the 4 nations of the UK, 1993-2017. Br. J. Clin. Pharmacol. 87 (8), 3349–3353. 10.1111/bcp.14727 33393673

[B31] Sills G. J. (2006). The mechanisms of action of gabapentin and pregabalin. Curr. Opin. Pharmacol. 6, 108–113. 10.1016/j.coph.2005.11.003 16376147

[B32] Smith R. V. Havens J. R. Walsh S. L. (2016). Gabapentin misuse, abuse and diversion: a systematic review. Addiction 111 (7), 1160–1174. 10.1111/add.13324 27265421 PMC5573873

[B33] Tardelli V. S. Bianco M. C. M. Prakash R. Segura L. E. Castaldelli-Maia J. M. Fidalgo T. M. (2022). Overdose deaths involving non-BZD hypnotic/sedatives in the USA: trends analyses. Lancet Reg. Health amer. 10, 100190. 10.1016/j.lana.2022.100190 36777690 PMC9904096

[B34] Torrance N. Veluchamy A. Zhou Y. Fletcher E. H. Moir E. Herbert H. I. (2020). Trends in gabapentinoid prescribing, co-prescribing of opioids and benzodiazepines, and associated deaths in Scotland. Br. J. Anaesth. 125 (2), 159–167. 10.1016/j.bja.2020.05.017 32571568

