# Supplementary material for: Incident gabapentin prescribing associated with opioid and benzodiazepine/Z-drug prescribing – a population-based longitudinal study in primary care
Source: Front Pharmacol. 2025 Aug 13;16:1583415. doi: 10.3389/fphar.2025.1583415 (PMC12381525; doi:10.3389/fphar.2025.1583415)
Supplement: Supplementary file 1 [file Table1.doc]

**Supplementary Table 1**. 18 chronic diseases (ICD-10) combined into one group of variables, called other conditions, used in the calculation of IRR for gabapentin.

| Disease | **ICD-10 code** |
| --- | --- |
| Tuberculosis | A15-A19 |
| Herpes zoster | B02 |
| Human immunodeficiency virus infection | B20-B24 |
| Thyroid diseases | E00-E07 |
| Diabetes | E10-E14 |
| Metabolic diseases | E65-E68 |
| Hyperlipidaemia | E78 |
| Cardiovascular disease | I00 – I09, I16 – I99 |
| Hypertension | I10 – I15 |
| Chronic obstructive pulmonary disease | J44 |
| Asthma | J45-J46 |
| Bronchiectasis | J47 |
| Gastro-oesophageal reflux | K21 |
| Psoriasis | L40 |
| Ankylosing spondylitis | M45 |
| Osteoporosis | M80-M82 |
| Other chronic musculoskeletal problems | M00 – M03, M20 – M43, M46 – M51,  M60 – M77, M83 – M99 |
| Renal disease | N18-N19 |
